# Wavelength Division Multiplexing-Based High-Sensitivity Surface Plasmon Resonance Imaging Biosensor for High-Throughput Real-Time Molecular Interaction Analysis

**DOI:** 10.3390/molecules29122811

**Published:** 2024-06-13

**Authors:** Zhenxiao Niu, Hao Du, Lin Ma, Jie Zhou, Zhengqiang Yuan, Ronghui Sun, Guanyu Liu, Fangteng Zhang, Youjun Zeng

**Affiliations:** 1 School of Physics and Optoelectronic Engineering, Guangdong University of Technology, Guangzhou 510006, China; 17793175617@163.com (Z.N.); 15536552597@163.com (H.D.); malin@gdut.edu.cn (L.M.); liuguanyulh@163.com (G.L.); zhang.ft@gdut.edu.cn (F.Z.); 2 Guangdong Provincial Key Laboratory of Sensing Physics and System Integration Applications, Guangdong University of Technology, Guangzhou 510006, China; 3 School of Laboratory Medicine, Hubei University of Chinese Medicine, 16 Huangjia Lake West Road, Wuhan 430065, China; zhoujiecomeon@163.com; 4 Hubei Shizhen Laboratory, Wuhan 430065, China; 5 School of Biomedical and Pharmaceutical Sciences, Guangdong University of Technology, Guangzhou 510006, China; yuanzq@gdut.edu.cn (Z.Y.); 2112312086@mail2.gdut.edu.cn (R.S.)

**Keywords:** surface plasmon resonance, optical biosensors, kinetic analysis

## Abstract

In this study, we report the successful development of a novel high-sensitivity intensity-based Surface Plasmon Resonance imaging (SPRi) biosensor and its application for detecting molecular interactions. By optimizing the excitation wavelength and employing a wavelength division multiplexing (WDM) algorithm, the system can determine the optimal excitation wavelength based on the initial refractive index of the sample without adjusting the incidence angle. The experimental results demonstrate that the refractive index resolution of the system reaches $1.77\times 10^{-6}$ RIU. Moreover, it can obtain the optimal excitation wavelength for samples with an initial refractive index in the range of 1.333 to 1.370 RIU and accurately monitor variations within the range of 0.0037 RIU without adjusting the incidence angle. Additionally, our new SPRi technique realized real-time detection of high-throughput biomolecular binding processes, enabling analysis of kinetic parameters. This research is expected to advance the development of more accurate SPRi technologies for molecular interaction analysis.

## 1. Introduction

The significance of analyzing intermolecular interactions lies in gaining deeper insights into the underlying mechanisms, structures, and functions between molecules [1,2,3]. Such knowledge holds value across many domains. For instance, in drug development, intermolecular interactions constitute critical steps between medications and their target proteins [4,5]. Through analyzing these interactions, binding patterns, affinities, and specificities between drugs and targets can be elucidated, thereby guiding optimization of drug design [6,7,8]. In biological research, intermolecular interactions form the foundation of vital processes. Examining such interactions serves to uncover structural and functional relationships between proteins, nucleic acids, and other biomolecules, leading to a more comprehensive understanding of biological regulations and dynamics [9,10,11]. Presently, techniques such as bio-layer interferometry (BLI), protein microarrays, and enzyme-linked immunosorbent assay (ELISA) are commonly employed to study and analyze intermolecular interactions [12,13,14]. However, these methods require preprocessing steps involving labeling, which can introduce complexity. They also have limited detection capabilities and throughput, precluding real-time monitoring of molecular interaction dynamics.

Compared to the analytical methods mentioned earlier, Surface Plasmon Resonance (SPR) sensing technology offers distinct advantages in terms of label-free and real-time detection capabilities [4,15,16,17]. One variation of this technology, called SPR imaging (SPRi) [18], allows for parallel detection of multiple samples, thereby enhancing detection efficiency [19,20,21]. Currently, four modulation modes have been proposed for SPRi sensing technology: phase-based, angle-based, wavelength-based, and intensity-based [22,23,24]. Phase-based SPRi sensing technology involves fixing the angle and wavelength of the incident light and modulating it, followed by demodulating the reflected light [25]. By analyzing the phase difference between the incident and reflected light, the sample can be detected [26,27]. This method boasts ultra-high sensitivity, typically with a dynamic range on the order of 10^−4^ RIU [28,29]. However, it does have limitations when there are refractive index differences between samples or non-uniform metal film thickness on the sensing chip, as the test range can exceed the system’s dynamic range. Consequently, this modulation method is not ideal for imaging detection. In comparison, angle modulation-based SPRi sensing technology offers the advantage of a larger dynamic range [30]. In this method, the incident light’s wavelength is fixed, and the incident angle is scanned. By analyzing the angular spectrum, the resonance angle of the sample can be determined, reflecting changes in the sample [31,32]. However, in SPRi systems based on prism structures, different incident angles can cause varying degrees of distortion, making it impossible to achieve in situ detection of the same spot. Hence, this method also has limitations for high-throughput imaging detection. Wavelength-based SPR sensing technology, similar to angle-based SPR sensing, falls under spectral sensing [33]. Its dynamic range and sensitivity are comparable to angle-based SPR [34]. In wavelength-based SPR sensing technology, the incident angle is fixed, and either the incident wavelength is scanned or the reflected spectrum is analyzed to obtain the SPR spectrum. This reflects changes in the sample through variations in the resonance angle. This method allows for flexible selection of the optimal excitation wavelength for different samples, providing unique advantages for imaging [35,36]. However, the response time of the spectrometer components reduces the system’s temporal resolution during wavelength scanning, which prevents tracking of molecular interaction processes in real-time. Furthermore, spectrometer components are typically complex and costly, which leads to high development costs and reduced system stability for wavelength-based SPRi sensing systems.

Compared to the aforementioned three modulation modes of SPRi, intensity-based SPRi sensing technology offers advantages of simple structure, easy implementation, and high temporal resolution [18,37,38]. It is the most commonly used modulation mode in commercial SPRi detection instruments. In this method, the incident wavelength and angle are typically fixed, and the reflected light intensity is directly monitored to sense the changes in the sample. However, this modulation mode has the following limitations: 1. Low sensitivity: Intensity-based SPRi sensing technology directly monitors the reflected light intensity, making it susceptible to fluctuations in the light source. This can result in lower sensitivity compared to other modulation modes. 2. Small dynamic range: This sensing method usually fixes the incident light angle and wavelength and then senses a linear range in the spectrum or angular spectrum curve. As a result, the dynamic range is typically on the order of 10^−3^ RIU [24]. These limitations should be taken into consideration when using intensity-based SPRi sensing technology for intermolecular interaction analysis. 

In order to enhance the sensitivity of the intensity-based SPRi sensing system, Zybin proposed a dual-wavelength difference method [39]. This method utilizes two specific excitation wavelengths to excite the SPR phenomenon and obtain corresponding reflected light intensities. By subtracting the two reflected light signals, the signal is amplified, and the system sensitivity reaches $5\times 10^{-6}$ RIU. It also enables parallel monitoring of 4-channel samples. Similarly, in our previous research, we also proposed an SPRi sensing technology based on the wavelength difference algorithm. We used incoherent light sources as the excitation wavelengths to avoid speckle noise from coherent light sources. Under imaging conditions, the sensitivity achieved was 2 × 10^−6^ RIU [40]. To address the issue of dynamic range, we proposed an intensity-based SPRi sensing technology based on a 4f structure [41]. By incorporating a 4f system in both the incident and reflected light paths, the central position of the sensing surface remains unchanged during angle adjustment. Although this method allows for the selection of optimal excitation angles for different samples to some extent, it still cannot avoid distortion caused by the prism during the angle adjustment process, making it unable to achieve in situ detection.

In this study, we proposed a robust intensity-based SPRi sensing system with high sensitivity and dynamic selection of optimal excitation wavelengths. A wavelength division multiplexing (WDM) algorithm was introduced to obtain the optimal excitation wavelengths for different samples in the intensity-based SPRi sensing mode, thereby enhancing the system’s sensitivity. Based on this algorithm, a sensing system was constructed using a white light LED combined with a filter wheel as the excitation light source, enabling the selection of optimal excitation wavelengths for different samples without the need for modulation of the incident angle. Experimental results demonstrated that the system achieved a high sensitivity of 10^−6^ RIU, enabling parallel, accurate, and real-time detection of multi-channel molecular interaction processes. Finally, using this system, real-time monitoring of antigen–antibody interactions in a label-free state was achieved, allowing for detection of different concentrations of the analyte as well as analysis of the biomolecular kinetics.

## 2. The Principle of the Wavelength Division Multiplexing (WDM) Algorithm

### 2.1. Selection and Optimization of Excitation Wavelength

**Figure 1:**
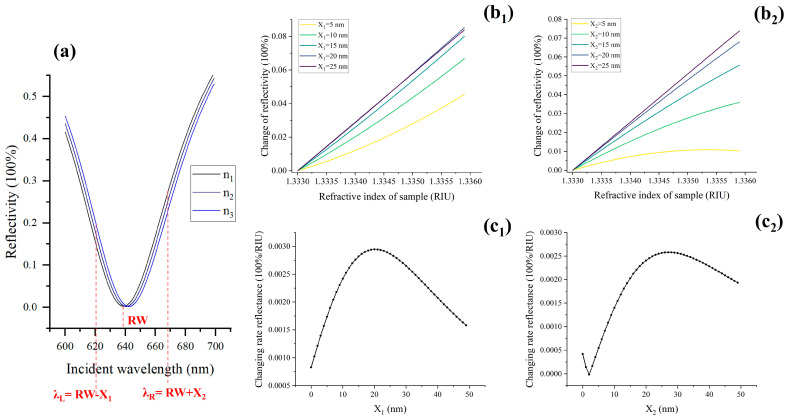
Theoretical analysis of selecting the optimal excitation wavelength. (**a**) SPR spectrum, RW: resonance wavelength, λ_L_ and λ_R_, are the excitation wavelengths on both sides of the resonance wavelength, respectively. (**b_1_**,**b_2_**) Reflectance variation curve with sample refractive index under different excitation wavelengths, b_1_ and b_2_, correspond to excitation wavelengths of λ_L_ and λ_R_, respectively. (**c_1_**) and (**c_2_**) are the curves of reflectivity variation with sample refractive index under different excitation wavelengths (λ_L_ and λ_R_), respectively.

In this section, we will examine the influence of the excitation wavelength on the system performance. When the refractive index of the sample changes, the corresponding SPR spectrum will shift. Sensing can be achieved by monitoring the intensity of reflected light at specific wavelengths. Since different samples may have optimal excitation wavelengths, we use the resonance wavelength (RW) as a reference for further analysis. To begin, we generate an ideal SPR spectral curve with a different refractive index (n_1–3_) based on the Fresnel formula, as depicted in Figure 1a. In this figure, λ_L_ and λ_R_ represent the excitation wavelengths on the left and right sides of the RW, respectively. The relationship between λ_L_, λ_R_, and RW can be expressed as λ_L_ = RW − X_1_; λ_R_ = RW + X_2_, where X_1_ and X_2_ are constants representing the offset between the excitation wavelength and the RW. Figure 1b_1_. illustrates the reflectivity curve with varying sample refractive indices at different excitation wavelengths λ_L_. It can be observed that there are differences in the slope and linearity of the curve under different excitation wavelengths. A steeper slope indicates a greater change in the SPR signal caused by the same refractive index change in the system, resulting in higher sensitivity. Similarly, Figure 1b_2_. demonstrates the SPR signal-refractive index variation curve at different excitation wavelengths λ_R_. As with λ_L_, there are differences in the curve characteristics under different excitation wavelengths λ_R_. By analyzing the curves shown in Figure 1b_1_,b_2_, we can determine the impact of excitation wavelength on the sensitivity and response characteristics of the system. This analysis helps in selecting the optimal excitation wavelength for specific samples and optimizing the system performance for accurate and reliable sensing.

In order to accurately determine the optimal excitation wavelength, we conducted a detailed simulation analysis on the values of X_1_ and X_2_, ranging from 0 to 50 nm with a step size of 1 nm. The curves of reflectivity with different refractive index values are illustrated in Figure 1c_1_,c_2_. From the simulation results, it can be observed that when X_1_ is set to 20 nm and X_2_ is set to 28 nm, the SPR signal exhibits the highest sensitivity to changes in refractive index. This indicates that the system achieves its maximum sensitivity at this excitation wavelength. Therefore, the optimal excitation wavelength can be determined as follows: λ_L_ = RW − 20 nm and λ_R_ = RW + 28 nm. 

**Figure 2:**
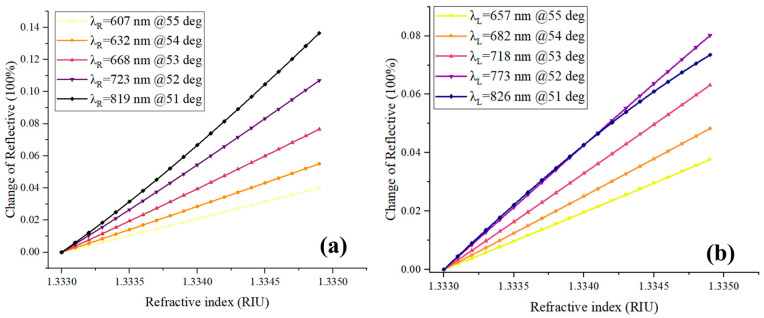
Curve of reflectivity variation with sample’s refractive index under different excitation wavelengths, the excitation wavelengths of (**a**) and (**b**) are λ_L_ and λ_R_, respectively.

In addition to the optimal excitation wavelength, the wavelength range in which the optimal excitation wavelength is located also impacts the sensitivity of the system. To further explore this relationship, we conducted simulations and discussed the influence of different excitation bands on the SPR signal in relation to the sample’s refractive index. We generated ideal SPR spectral curves for various samples with different refractive indices using the Fresnel formula. We then fixed the excitation wavelengths at λ_L_ = RW − 20 nm and λ_R_ = RW + 28 nm, respectively, and obtained the reflected light intensity signals. Next, we varied the incident angle to place the λ_L_ or λ_R_ in different wavelength bands within the visible to near-infrared range. The sensing signal curves of the excitation wavelengths (λ_L_ and λ_R_) within the visible to near-infrared wavelength range are depicted in Figure 2a,b. The results clearly demonstrate that the excitation wavelength within the near-infrared band yields a larger response signal for the same sample. Thus, utilizing the near-infrared band as the excitation wavelength provides higher sensitivity for the system. This analysis emphasizes the importance of selecting the appropriate wavelength range for excitation to optimize the sensitivity of the system in SPR sensing applications.

### 2.2. WDM Algorithm

By utilizing multi-wavelength incident light excitation, it becomes possible to flexibly select the optimal excitation wavelength for different samples without adjusting the incident angle. This allows for parallel detection of multiple samples. Considering that the excitation band of incoherent broadband light sources, such as white LEDs or halogen lamps, is usually from the visible light band to the near-infrared band, and the near-infrared band produces a greater signal response to the same sample, it can be beneficial to optimize the system for near-infrared excitation wavelengths. In the following discussion, we will use the fixed calculation parameters for the optimal excitation wavelength, specifically X_1_ = 20 nm and X_2_ = 30 nm. Based on the data from Figure 1c1, the optimal value for X_2_ is determined to be 28 nm. However, when selecting 30 nm, the difference in reflectivity for the same sample is minimal, less than 1%, and can be considered negligible. Therefore, for the sake of convenience, we will focus on analyzing and discussing the incident wavelengths of the six near-infrared bands, specifically 750, 770, 790, 810, 830, and 850 nm.

**Figure 3:**
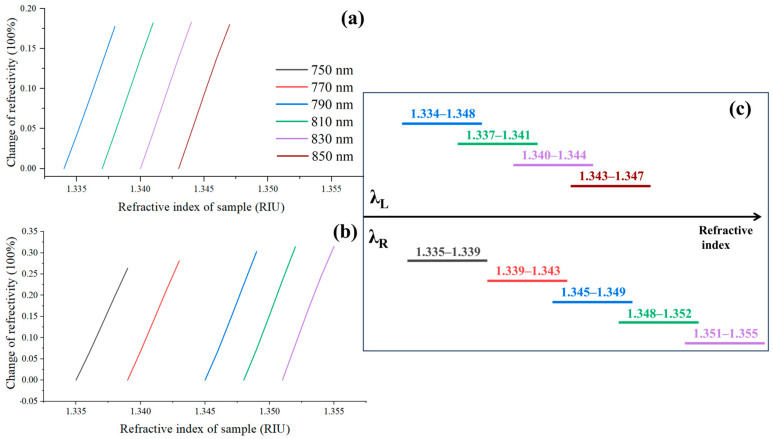
Linear regions corresponding to different excitation wavelengths. (**a**) and (**b**) is the linear regions corresponding to excitation wavelengths λ_L_ (790, 810, 830 and 850 nm) and λ_R_ (750, 770, 790, 810, and 830 nm), respectively; (**c**) Summary plot of six linear regions with excitation wavelengths.

Based on the analysis in Section 2.1, once the RW is determined, there are two optimal excitation wavelengths, λ_L_ and λ_R_, corresponding to two linear regions that can be utilized for sensing. We set samples with refractive indices ranging from 1.333 to 1.355 RIU, with a step size of 0.001 RIU. Within this range, λ_L_ can be set at 790, 810, 830, and 850 nm, with each wavelength having a corresponding linear range, as shown in Figure 3a. Similarly, λ_R_ can be selected at 750, 770, 790, 810, and 830 nm, with their respective linear ranges illustrated in Figure 3b. By examining Figure 3a,b, it can be observed that each excitation wavelength has a dynamic range of 0 to 0.004 RIU. The linear regions corresponding to the six excitation wavelengths are clearly depicted in Figure 3c. From this figure, it is evident that precise detection can be achieved by dynamically selecting six excitation wavelengths when the initial refractive index of the sample falls within the range of 1.334 to 1.350 RIU and with a variation in 0.004 RIU.

According to the theoretical analysis presented above, the process of the WDM algorithm in this article can be described as follows: (1) The first step involves acquiring the RW of the sample under consideration; (2) Next, the optimal excitation wavelengths λ_L_ and λ_R_ are determined based on the RW obtained in the previous step; (3) Subsequently, the incident wavelength provided by the system is compared with the available excitation wavelengths, and the closest wavelength is selected as the excitation wavelength. By selecting the excitation wavelength appropriately, it is possible to achieve accurate sensing without the need to adjust the incident angle.

## 3. System

**Figure 4:**
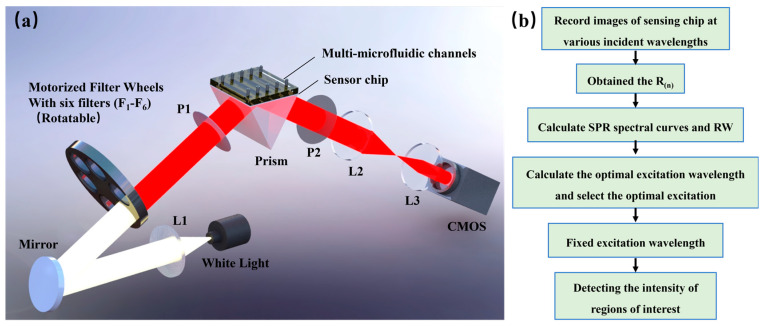
The SPRi system. (**a**) Optical path diagram. P1, P2, polarizer; L1–L3, lens; F_1_–F_6_, filter. (**b**) System workflow diagram.

The optical path of the system is illustrated in Figure 4a. It begins with a white LED (LB-SPL-20W, power: 3 W, LBTEK, Shenzhen, China) emitting broad spectrum white light, which is then collimated by lens L1. The collimated light passes through a reflector and enters the filter wheel. The wheel consists of six specific center wavelength filters (F_1_–F_6_) with center wavelengths of 750, 770, 790, 810, 830, and 850 nm, each with a full width at half height of 10 nm (198724, 198727, 198733, 198735, 198741, 198743; Grand Unified Optics, Beijing, China). The narrowband light that passes through the filter is directed towards the sensing module via polarizer P1. The sensing module comprises a prism, a sensing chip, and a flow cell. When the incident light is coupled with the prism, it excites the Surface Plasmon Resonance (SPR) phenomenon at the interface of the sensing chip and the sample. After passing through polarizer P2 and the imaging lens group (L2, L3) in sequence, the reflected light is received by the area array detector CMOS (DMK 33GP031, Imaging Source, Bremen, Germany). This detector captures the intensity distribution of the reflected light for further analysis and sensing of the sample.

The system workflow, as depicted in Figure 4b, consists of two main processes: excitation wavelength selection and detection. During the excitation wavelength selection process, the filter wheel rotates sequentially from positions F_1_ to F_6_. At each stop, the wheel stabilizes, and the CMOS camera automatically captures an image of the sensing surface, recording the acquired data. Once a full scanning cycle is completed, the reflectance values (R(n)) are obtained for different incident wavelengths using Formula (1). Additionally, the average intensity of the SPR region (I_SPR(n)_) and the background (I_No-SPR(n)_) is calculated, with ‘n‘ representing the number of filters. Polynomial fitting of the reflectance values R_(n)_ (where n ranges from 1 to 6) is performed to derive the SPR spectral curve and determine the RW.

(1) R(n)=ISPR(n)INo−SPR(n)

The optimal excitation wavelength is next calculated using the formulas λ_L_ = RW − 20 nm and λ_R_ = RW + 30 nm. The six incident wavelengths are subtracted from λ_L_ and λ_R_ and the absolute differences evaluated, with the minimum difference identifying the selection for that time point. Following wavelength selection, the system adjusts to the corresponding filter position, allowing the CMOS camera to automatically acquire images and monitor light intensity changes within any region of interest. And the time resolution of the system is about 0.3 s.

## 4. Results and Discussion

### 4.1. Materials and Chemicals

Materials: The sensor chip in the system is based on glass 1 mm thick (Length: 18 mm, width: 18 mm) as the substrate, with a 48 nm thick gold layer being deposited on the substrate through evaporation, and a 2 nm chromium layer of spacing between the glass substrate and the gold coating to increase the fixation effect. The prism is an equilateral triangle with a side length of 18 mm made by SF11 (1.785 RIU). The transmittance of the 750, 770, and 790 nm wavelengths are >50%, and the transmittance of the 810, 830, and 850 nm wavelengths are >45%. The extinction ratio of the polarizer is 1000:1.

Chemicals: The goat anti-human IgG, goat anti-rabbit IgG, and human IgG, bovine serum albumin (BSA) and phosphate-buffered saline (PBS) were obtained from Solarbio Science & Technology Co., Ltd. (purity: ≥99.5%, Beijing, China). Refractive index matching oil (1.780 RIU) was bought from Cargille Laboratories, Inc. (Cedar Grove, NJ, USA). NaCl was obtained from Aladdin (purity: ≥99.5%, Shanghai, China). 

### 4.2. System Performance Testing

The sensitivity of the system and the ability to flexibly select the optimal excitation wavelength are demonstrated in this section. We tested the performance of the system by monitoring the NaCl solution with different concentrations. The test solution is divided into five groups, with initial concentrations of 0, 5, 10, 15, and 20% (corresponding refractive index: 1.3330, 1.3423, 1.3515, 1.3608, and 1.3700 RIU). Then, each group of test solutions increases the concentration by 0.5% four times, on the basis of the initial concentration as the next test solution. For example, starting with a concentration of 0%, we sequentially introduce NaCl solutions with concentrations of 0.5%, 1%, 1.5%, and 2%; when the initial concentration is 10%, NaCl concentrations of 10.5%, 11%, 11.5%, and 12% are sequentially introduced. The refractive index change corresponding to a 2% concentration change is 0.0037 RIU.

**Figure 5:**
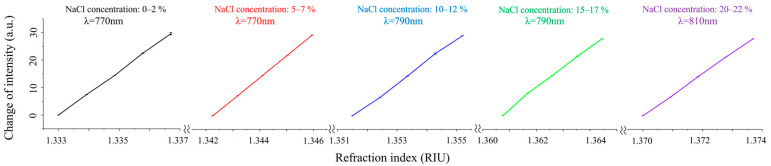
SPR signal shift versus refractive index at different optimal excitation wavelengths.

Five experimental groups were conducted independently of each other. During the experiment, five portions of the test solution from each group were sequentially passed into the flow cell from high to low concentration, and the system recorded the SPR signal response caused by different concentrations of NaCl. The detection results of the system on the five groups of samples mentioned above are shown in Figure 5. From the results, it can be seen that different initial refractive indices correspond to different optimal excitation wavelengths, which are calculated by the WDM algorithm. And each group of reflected light intensity changes exhibits good linearity and consistency with the change in refractive index of the sample at the optimal excitation wavelength. For the same group, we repeated each experiment three times, and in all experiments, the maximum error was less than 1%. For all groups, the deviation of the intensity signal variation was less than 4%. This proves the sensing performance of the system for different samples, and it has good consistency and repeatability.

(2) $$\sigma_{RI}=\frac{\sigma_{n}}{\sigma_{I}}{\Delta \sigma }_{SD}$$

The refractive index resolution (RIR) of the system is another important parameter. According to Formula (2) and by employing the average values of data from the five groups presented in Figure 5, the RIR of our system is calculated as $\sigma_{RI}=1.77\times 10^{-6}$ RIU, where $\sigma_{n}=0.0037$ RIU is the change of refraction index, and $\sigma_{I}=29.26$ a.u. is the corresponding intensity response. The ${\Delta \sigma }_{SD}=$0.014 RIU is the root mean square of noise, which is calculated by conducting a 10 min baseline test with the sample of pure water.

### 4.3. Detection of Antibody-Antigen Binding

Before starting testing, we needed to preprocess the sensing chip, which involved three steps: (1) PBS (0.01 M, pH = 7.3) for 5 min, then introduce human IgG (100 μg/mL); the antigen would be fixed on the chip surface through physical adsorption as a biomolecular probe [35] After signal stabilizing (about 10 min), introduce PBS again for 10 min; (2) Introduce BSA (100 μg/mL) to block nonspecific binding sites and avoid signal changes caused by nonspecific binding; (3) Connect PBS again to clean the chip for 10 min. 

**Figure 6:**
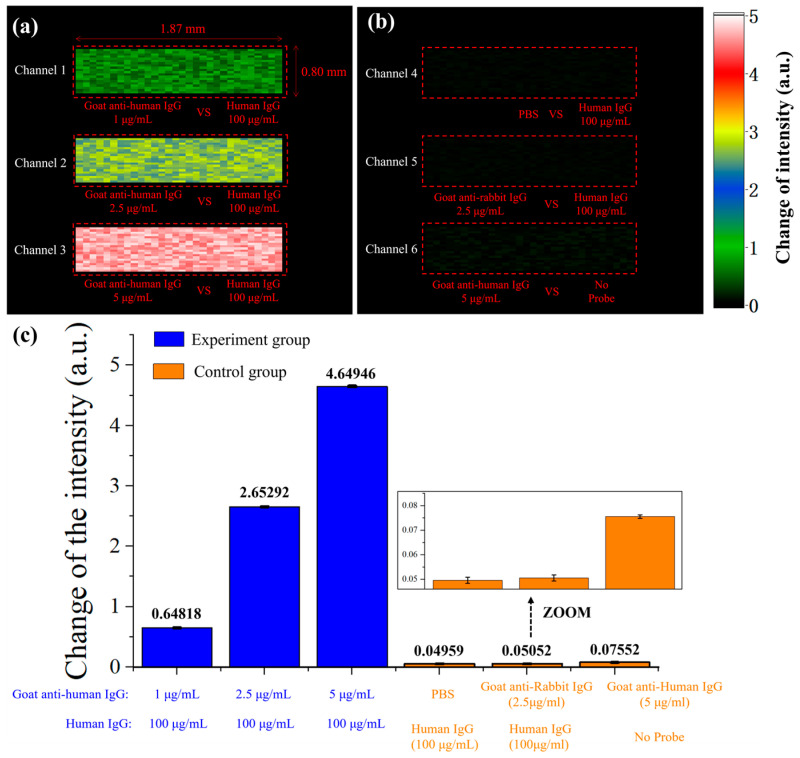
The images of shift in intensity. (**a**) Experimental group: the samples introduced in the three channels from left to right are 1, 2.5, and 5 μg/mL goat anti-human IgG. (**b**) Control group: the surface probes of the test liquid and sensing chip are human IgG and PBS (channel 4), goat anti-rabbit IgG and human IgG (channel 5), goat anti-human IgG and no probe molecules (channel 6). (**c**) The average change in SPR signal after three repeated experiments on experimental and control groups.

After processing the sensing chip, we began the testing experiment. Firstly, PBS is carried out and the SPR signal is monitored in real-time. After the signal stabilized, goat anti-human IgG was introduced at concentrations of 1, 2.5, and 5 μg/mL into three channels (channels 1–3), respectively. After about 5 min, PBS was introduced again. In the above steps, the flow rate for all solutions introduced was 20 μL/min. The SPRi scheme allowed us to obtain the 2D intensity difference in each pixel before and after the reaction of the sensing surface. As illustrated in Figure 6, the color of each pixel in the figure represents the intensity shift on the sensing surface before and after the reaction. Figure 6a shows the antigen–antibody reaction channels, in which goat anti-human IgG specifically binds to human IgG, causing changes in the SPR signal, and the signal increases with the increase of antibody concentration. 

In addition, we conducted three sets of comparative experiments, and the results are shown in Figure 6b. In channel 4, the surface probe of the chip is human IgG, the test solution is PBS, and there are no antibodies in the test solution; in channel 5, the surface probe of the chip is also human IgG, and the test solution is goat anti-rabbit IgG (2.5 μg/mL). Channels 1–2 show no obvious signal changes. These two experiments demonstrate that there is no significant change in the SPR signal in the absence of specific protein molecules in the test solution. In channel 6, there are no probe molecules on the surface of the chip, and the test solution is goat anti-human IgG (5 μg/mL). This channel also shows no significant signal changes, indicating that when there are no probe molecules on the chip surface, the presence of antibody molecules in the test solution cannot cause SPR signal changes. These results demonstrate that only antigen–antibody-specific binding can cause changes in SPR signals, validating the specific molecular detection capabilities of our system.

We repeated each experiment for both the experimental and control groups three times, and the average signal change before and after the reaction is depicted in Figure 6c. As shown in the figure, the signal change in the experimental group is significantly greater than that in the control group. Furthermore, the experimental results demonstrate that the system exhibits good repeatability. The inset in the figure provides an enlarged image of the control group (PBS vs. human IgG), and the error deviation for each group of experiments is less than 1%. Furthermore, the limit of detection (LOD) of our platform was calculated using the following equation [3,35], where C represents the lowest concentration of the analyte, R denotes the sensor response corresponding to the lowest concentration of analyte binding with its ligand, and $\sigma_{SD}$ is the root-mean-square (RMS) noise of our platform. For our platform, these three parameters are 1 μg/mL, 0.64818 a.u., and 0.03772 a.u., respectively. Consequently, the LOD of our platform is 0.0581937 μg/mL.

(3) $$LOD=\frac{C}{R}\times \sigma_{SD}$$

**Figure 7:**
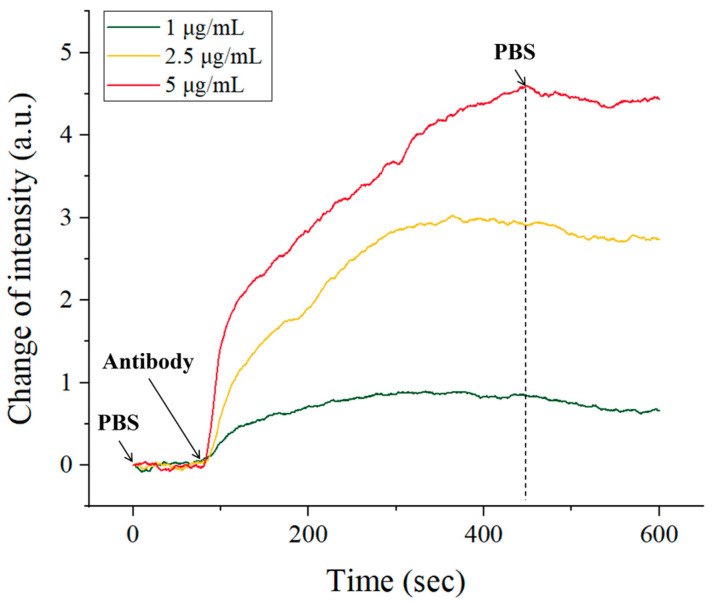
Real-time intensity response of human IgG (100 μg/mL) and goat anti-human IgG (1, 2.5, and 5 μg/mL) binding reactions.

In addition, our system can achieve real-time monitoring of the biomolecular interaction process. The antigen–antibody interaction is shown in Figure 7. The three curves represent the SPR response signals of the real-time reaction between goat anti-human IgG and human IgG at different concentrations in the experimental group. As the concentration increases, it causes larger signal changes and a greater difference before and after the reaction. Based on the respond curve, we can also analyze the kinetic parameters of molecules by a first-order kinetic formula [4,42]. $R_{max}$ is the maximum SPR signal at saturation of the reactant; dRdt is the derivative of the SPR signal with respect to time t, which is the rate at which antigen molecules fixed on the surface of the sensing chip combine with antibodies to form complexes; c is the mass concentration of the analyte; $k_{a}$ and $k_{d}$ are binding constants and dissociation constants, respectively. Calculated results: $k_{a}\approx 1.33\times 10^{5}\;M^{-1}\cdot s^{-1}$; $k_{d}\approx 7.47\times 10^{-3}\;s^{-1}$, and the molecular affinity constant can be calculated from KD=kdka≈5.62×10−8M. The above calculation results are comparable to those reported in the other literature [42,43], demonstrating the system’s capability for biomolecular dynamic analysis.

(4) $$\frac{dR}{dt}=k_{a}cR_{max}-(k_{a}c+k_{d})R$$

## 5. Conclusions

In summary, we developed a WDM-based high-sensitivity SPRi biosensor. The system achieved acquisition of the optimal excitation wavelength for different samples without adjusting the incident angle, further enabling parallel detection of multi-channel samples. The experimental results demonstrate that the refractive index resolution of the system achieves 1.77 × 10^−6^ RIU, which is currently the highest resolution reported for an intensity-based SPRi system. Importantly, the system has the capability for parallel detection of multiple specific molecular concentrations and can analyze molecular dynamics parameters through real-time response curves without requiring any specific staining or pretreatment of molecules. This provides an efficient, convenient, and highly sensitive means of detecting specific molecular interactions and studying biological processes. Nevertheless, opportunities remain to optimize its sensing performance. Potential approaches include utilizing narrower bandwidth filters, employing a complementary metal-oxide-semiconductor detector with a higher signal-to-noise ratio, or utilizing higher intensity incoherent broadband light sources.

## Data Availability

No new data were created or analyzed in this study. Data sharing does not apply to this article. We used only publicly available datasets for experimentation.
